# Browsing Isolated Population Data

**DOI:** 10.1186/1471-2105-6-S4-S17

**Published:** 2005-12-01

**Authors:** Gianmaria Mancosu, Massimiliano Cosso, Francesca Marras, Cesare Cappio Borlino, Giuseppe Ledda, Teresa Manias, Mauro Adamo, Donatella Serra, Paola Melis, Mario Pirastu

**Affiliations:** 1 SharDNA Life Sciences, Cagliari, Italy; 2 Istituto di Genetica delle Popolazioni, CNR, Alghero (Sassari), Italy

## Abstract

**Background:**

In our studies of genetically isolated populations in a remote mountain area in the center of Sardinia (Italy), we found that 80–85% of the inhabitants of each village belong to a single huge pedigree with families strictly connected to each other through hundreds of loops. Moreover, intermarriages between villages join pedigrees of different villages through links that make family trees even more complicated. Unfortunately, none of the commonly used pedigree drawing tools are able to draw the complete pedigree, whereas it is commonly accepted that the visual representation of families is very important as it helps researchers in identifying clusters of inherited traits and genotypes. We had a representation issue that compels researchers to work with subsets extracted from the overall genealogy, causing a serious loss of information on familiar relationships.

To visually explore such complex pedigrees, we developed PedNavigator, a browser for genealogical databases properly suited for genetic studies.

**Results:**

The PedNavigator is useful for genealogical research due to its capacity to represent family relations between persons and to make a visual verification of the links during family history reconstruction. As for genetic studies, it is helpful to follow propagation of a specific set of genetic markers (haplotype), or to select people for linkage analysis, showing relations between various branch of a family tree of affected subjects.

**Availability:**

PedNavigator is an application integrated into a Framework designed to handle data for human genetic studies based on the Oracle platform. To allow the use of PedNavigator also to people not owning the same required informatics infrastructure or systems, we developed PedNavigator Lite with mainly the same features of the integrated one, based on MySQL database server. This version is free for academic users, and it is available for download from our site

## Background

Acquiring, storing and manipulating genealogical information is an important issue in human genetic research. In our studies of genetically isolated populations in a remote mountain area in the center of Sardinia (Italy) called Ogliastra [1], we had the possibility of reconstructing back to the XVI century the pedigree for most of the inhabitants of the small villages located in that region. Despite the fact that the villages are nowadays characterized by less than 1,000 inhabitants, the overall population we are, in principle, able to consider for analysis, including living individuals and their ancestors, is about 10,000 persons per village. By genealogic reconstruction, we found that 80–85% inhabitants of each village belong to a single huge pedigree with families strictly connected to each other through hundreds of loops. Moreover, intermarriages between villages join pedigrees of different villages through links that make family trees even more complicated.

When the descent of so many people is being investigated, the problem of how to handle huge pedigrees arises, as genealogies taken into account can be composed of up to thousands of individuals and typically include loops, multiple mates and several related families. The problem is how to give a clear picture of family relationships by means of a graphical representation in order to highlight internal organization of pedigrees that is also easy to understand. To address this point, researchers usually deal with pedigrees using software like: Cyrillic, Cherwell Scientific, 2000; Progeny, Progeny Corp, 2000; GAP, Epicenter Software, 2000; CoPE, GIS Infobiogen, 1999 [2]. For a more complete list, see [3]. These programs are suited to show structures of quite complex and large family pedigree as well and they allow the association of properties to each person, like phenotypes and genotypes. To our knowledge, none of the programs mentioned above is capable of drawing the whole pedigrees we are faced with, raising a representation issue and compelling researchers to work with subsets extracted from the overall genealogy. Unfortunately, because of typical characteristics of villages, splitting pedigrees into smaller sub-units is not an easy task because information on kinship and inbreeding could be lost.

In order to give researchers a suitable support for navigating complex genealogical relations, we implemented a web-based application called PedNavigator (from a contraction of "Pedigree Navigator") -designed "ad hoc" to facilitate such a difficult task. With PedNavigator a user can easily navigate pedigrees by simply clicking on the desired person to expand branches of her/his pedigree. In this way, navigation becomes an easy task also with extensive pedigrees. The application is integrated with our Oracle database, giving researchers the opportunity of enriching each person's pedigree with the corresponding demographic, genetic, clinical and phenotypic data. A previous version of PedNavigator is depicted in [4], whereas in this paper we describe improvements of the new version: advanced graphic layout algorithms, addition of several navigation modes, graphical representation of up to four pathologies, status, kinship, genotypes and haplotypes.

## Methods

### General application description

**Figure 1:**
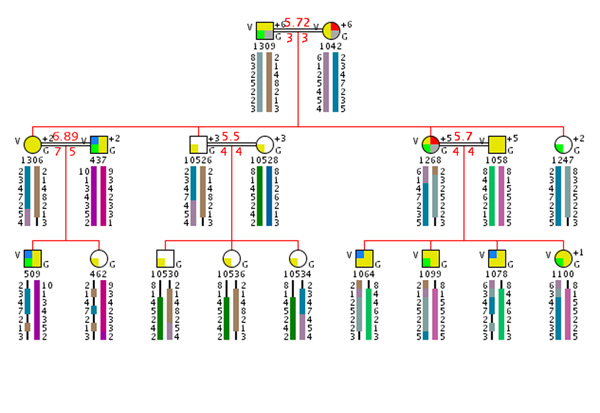
**PedNavigator main page example **Symbols with colored slices are affected persons, yellow slices show non-affected and white symbols stand for unknown status. Symbols are labeled with selected marker alleles. Plus signs near symbols indicate persons with expandable links. Red numbers are related with number of meiotic steps between consanguineous partners and their common ancestors (see text).

**Figure 2:**
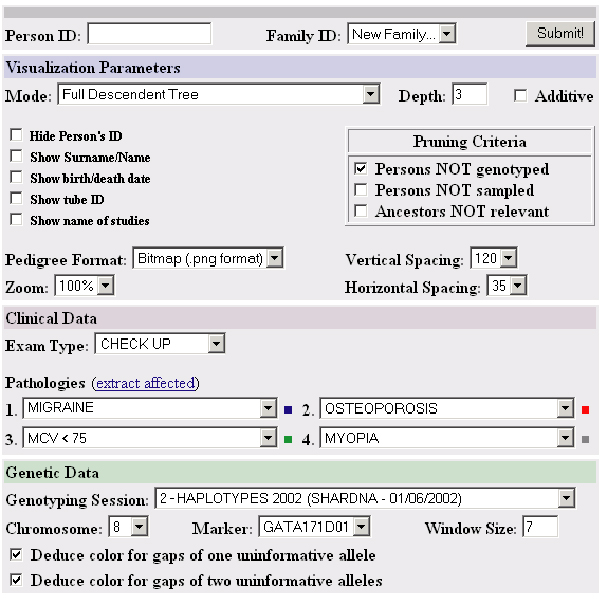
**PedNavigator main menu **The main menu is a web form divided into separated panel. The Visualization panel contains settings about visit modes, pruning criteria, zoom level and output format. The Clinical Data panel is designed to select up to four pathologies or traits, associated with persons, coloured according to the small square near the select box. The Genetic Data panel permits to choose markers and chromosomes to draw under individual's symbols.

PedNavigator can be installed on a web server (like Apache Tomcat) and can be consulted through Internet browsers supporting DHTML. The main page of PedNavigator is divided into two parts: (i) the upper side contains a browsable graphical representation of a pedigree (see Figure 1), whereas (ii) the lower part contains the main menu (see Figure 2), useful for setting up some relevant parameters, such as the navigation mode or the kind of information the user is interested in. For example, PedNavigator can either render pedigrees in anonymous form (only showing a numerical code for each person), as in Figure 1, or produce demographic information (like name, surname, date of birth, and date of death), not shown. In addition, taking advantage of the integration with genotypic/phenotypic database, it is possible to select genetic markers under investigation and also to show which individuals are actually affected by a given pathology, as shown in Figure 1. It is possible to show up to four pathologies at the same time.

To make the navigation easier, limiting the number of persons represented at the same time, a little "+", followed by the number of person's children, is drawn near the person's symbol, to indicate that the pedigree continues throughout that person towards subsequent generations.

From an architectural perspective, the application is composed by two sub-systems: the Query Sub-System, devised to query the genealogical database, together with the Layout Sub-System (internally called *PedPainter*), entrusted with actually drawing the pedigree. Both sub-systems are illustrated below in greater detail.

### Genealogic database query sub-system

The simple user interface described above allows an individual to be easily selected, by simply clicking on its symbolic representation. Since pedigree can become very complex and difficult to represent, database extraction algorithms must be configurable in order to follow only relevant relationships. In PedNavigator nine visit methods have been implemented:

(1) Snowflake (SF)

(2) Snowflake with Sibs (SFWS)

(3) Direct Ancestors (DA)

(4) Full Descendant Tree (FDT)

(5) Direct Paternal Descendants (DPD)

(6) Direct Maternal Descendants (DMD)

(7) Minimum Common Ancestor (MCA)

(8) Minimum Common Ancestor Paternal Lineage (MCAPL)

(9) Minimum Common Ancestor Maternal Lineage (MCAML)

The SF visit mode recursively includes all persons directly related with the person being investigated, by following downward and upward relationships until a given depth is reached, like a snowflake where the centre is the selected person. The SFWF is similar but with the addition of sibs of the extracted persons. The DA mode acts according to the classic pedigree representation, in which the ancestors of an individual are shown. The FDT mode supplies the descendants of an individual, including mates of everyone, if present. The DPD and DMD modes follow descendants according to paternal or maternal lineage, and is often used in genealogical studies to inquire on the spread of the surname or to support the Y-chromosome and mitochondrial DNA studies. The MCA builds a pedigree showing the minimum path linking two selected individuals, where founders are their minimum common ancestors. The MCAPL and MCAML are variants of MCA and follow paths containing only maternal or paternal lineages. It is worth pointing out that, for each visit mode, a search depth can be specified -in order to limit the number of persons included in a pedigree's representation and to increase its graphical clarity. As depicted in Figure 2, it is possible to apply some pruning algorithms to the pedigree extracted, with the aim to draw only relevant individuals: e.g. ones can exclude persons not typed because they do not add any further information for genetic analyses.

### Layout sub-system

The layout sub-system is devoted to graphically represent pedigrees extracted by the previous sub-system. As shown in Figure 1, it produces a bitmap using standard pedigree symbols (following [5]): a square for a male and a circle for a female, a dark filled colour for affected status, a yellow one for not affected, and a white one for unknown status. A black straight line between partners represents weddings (double line indicates consanguinity), and a four-point orthogonal poly-line joins sons with parents. Every family belonging to the same generation has links shown with different colours, to prevent misinterpretations in the presence of intersections. To improve clarity, it is possible to set layout zoom level and to specify distances between symbols.

A pedigree is internally represented by a directed graph: an abstract data type defined by a set of nodes and arcs, useful for modelling hierarchical networks. In particular, nodes (vertices) represent individuals, whereas arcs (edges) represent family ties. Provided that arc orientations are suitably chosen, any graph obtained from a pedigree is acyclic; thus, pedigrees can be suitably represented by hierarchical directed acyclic graphs (DAGs) and we can use algorithms taken from graph theory for handling pedigrees.

In the literature, different algorithms have been proposed to draw hierarchical DAGs. The underlying heuristics is usually devoted (i) to minimize the intersections between arcs, and (ii) to distribute nodes homogeneously. The complexity of the problem being NP-hard, we implemented a layout algorithm according to Sugiyama *et al. *[6]. This algorithm tackles the overall problem by performing a suitable decomposition into independent sub-problems, to be solved separately. In particular: (1) nodes are first arranged into levels (ranks), (2) then, each rank is locally ordered, trying to minimize edge crossing, (3) finally, nodes are spatially distributed, so that the average arc lengths and bends can be suitably minimized. The final scope of this algorithm is disposing each node in a virtual grid, where Y-axis is generation level and X-axis is the person's position within a level.

As for the first step, the natural order of nodes between genealogical generations promotes an efficient separation of vertices in separate ranks. In this case, some additional constraints hold: e.g., a daughter / son must belong to a generation inferior than the one of her / his parents and the siblings must belong to the same generation. As a result, every node is assigned to a non-ambiguous Y position within the virtual grid.

The second step highly affects the quality of a pedigree layout. Due to its intrinsic complexity the second step yields sub-optimal solutions, as suitable heuristics must be adopted to reduce the computational effort and time. In order to do that, we implemented some procedures described by Gansner *et al. *[7,8]. Originally designed for general graphs, we adapted them for handling genealogical graphs. Once nodes have been divided into ranks and local ordering has been performed, the third step of the algorithm assigns to each node a value in the X-axis of the virtual grid, to shorten node-to-node connections. To perform this task, we use a variant of the Sugiyama's algorithm, as described in [7]. Before generating the pedigree bitmap, positions X and Y of every node are transformed from relative-to-the-grid coordinates to absolute coordinates regarding the bitmap. Here we use an algorithm, of complexity *O(n)*, that applies scale factors to regulate total dimensions of the image, minimizing superimpositions of the text associated to each node. The resulting bitmap can now be inserted in the HTML page that represents the PedNavigator user interface.

### Implementation details

We implemented PedNavigator according to the client / server model; i.e., clients carry out requests to the web application server where the PedNavigator is installed, which in turn interrogates the database server. Following the principle of maximum portability, we wrote PedNavigator entirely in Java, also taking advantage of the Servlet technology, widely acknowledged as the "state of art" for dynamic web page generation. The Apache Tomcat Application Server, available under the GPL license for most operating systems, has been adopted as the web application server. To access the genealogical database, we adopted the standard JDBC Java package, which is an abstraction layer devised to perform SQL queries independently from the database vendor. On the other hand, while remaining compatible with the most widely used web browsers, the client side of the user interface takes advantage of small JavaScript scripts aimed at improving the quality of the user interface.

## Discussion

Our research is focused on the isolated population of several villages of Ogliastra because they present extremely advantageous characteristics for complex-trait studies. Our approach is family-based and the person is the key, but we must confront some daunting problems in our effort to fully use the data. The existence and accessibility of both ancient and recent archives (i.e. municipality registers, church archives, personal interviews) lets us reconstruct, in principle, the complete genealogy of the entire population of each village. This is an important prerequisite to build large genealogies which connect many selected individuals, chosen according to their phenotypes.

We needed something intuitive yet powerful to allow us to fully use this unprecedented wealth of genealogical data. Thus, we created PedNavigator to easily represent deep-rooted complex pedigrees with which the researcher can interact and better explore links between individuals to find even distant relationships. This intuitive informatics tool allows us to reconstruct, with an excellent degree of accuracy, genealogies of the villages since the early 1600's. What's more, our genealogies have been cross-validated with the study of mitochondrial DNA and the Y chromosome, identifying also ancient founders and their progeny [9]. Thanks to the completeness of the genealogies, we can calculate the kinship and find the common ancestors of virtually any pair of people in the database. In Figure 1, the red numbers over marriage links are a measure of the kinship between parents, calculated as the absolute values of the logarithm in base 2 of the kinship coefficient -it can be seen as a number of "equivalent" meiotic steps between consanguineous partners.

## Conclusion

PedNavigator has been used in the first phase of the genealogical data collection, to perform a visual verification of existing ties between individuals. In this way, the data entry activity has been greatly facilitated, with particular reference to the activity of checking whether the family ties between individuals have been correctly entered or not. In a subsequent phase, it has been used to follow the propagation of a selected haplotype throughout parenthood ties, so that possible genotypic mistakes can be easily located. Currently, thanks to further visualization options (e.g., the ability of showing phenotypic and genotypic values), PedNavigator has the capacity to select individuals for linkage analysis, showing relations (within various branches of a family tree) among affected subjects.
